# Editorial: Transcriptional Regulation in Cancers and Metabolic Diseases

**DOI:** 10.3389/fendo.2015.00173

**Published:** 2015-11-05

**Authors:** Wen Zhou

**Affiliations:** ^1^Department of Biological Sciences, Columbia University, New York, NY, USA; ^2^Braman Family Breast Cancer Institute, University of Miami Sylvester Comprehensive Cancer Center, University of Miami Miller School of Medicine, Miami, FL, USA; ^3^Molecular Oncology Research Program, Division of Surgical Oncology, Dewitt Daughtry Department of Surgery, University of Miami Miller School of Medicine, Miami, FL, USA

**Keywords:** p53, nuclear receptor, cell cycle, cancer stem cell, epigenetics, microRNA, exosome, transcriptional regulation

I am privileged to edit this Research Topic, *Transcriptional Regulation in Cancers and Metabolic Diseases* under the guidance of Dr. Carol Prives. We hereby thank Drs. Antonino Belfiore and Claire Perks for giving us the opportunity of editing the Research Topic. Given the large number of recent publications on transcriptional regulation, this Research Topic is timely needed. It covers many research hotspots, including p53’s gain-of-function mutation, p63’s role in epithelial cells, mitosis- or senescence-related transcription, and cancer-specific exosome. Overall, this *Research Topic* reviews and updates the current trends in transcriptional regulation.

Two *Mini Reviews* summarize the roles of heat shock proteins in transcriptional regulation (1, 2). In the first *Mini Review*, Alexandrova and Marchenko focused on a heat shock protein HSF1 in mutant p53 (mutp53)-mediated oncogenic activation. They highlighted a novel connection between HSF1 and mutp53 and hypothesized that pharmaceutically disrupting HSF1-mutp53 cooperation might be beneficial to cancer patients. In the second *Mini Review*, Khurana and Bhattacharyya outlined mechanisms through which another heat shock protein HSP90 activates gene expression. That is, HSP90 co-activates transcription factors, interacts with chromatin remodeling factors, and evicts histones from certain gene promoters.

Three *Reviews* explain transcriptional regulation of fate-determining transcription factors or cofactors (3–5). In the first *Review*, Yoh and Prywes illustrated the regulatory network that impinges upon the key epithelial transcription factor p63. In the second *Review*, Yoo and colleagues focused on a ubiquitin-fold modifier, UFM1 in breast cancer. In the third *Review*, Sun and colleagues categorized lysine acetyltransferases in normal and abnormal development of blood cells. Notably, Sun et al. reported current drug developments in lysine acetyltransferase inhibitors.

Two *Original research articles* evaluate transcriptional regulation in uveal melanoma and aging, respectively (6, 7). In the first *Original research article*, Huffman, Carstens, and Martinez profiled the expression levels of 48 human NRs across a panel of cell lines from uveal melanoma, cutaneous melanoma and melanocytes. In addition, the NR-to-NR and NR-to-genome expression correlation analyses identified RXRγ as a potential driver for melanoma-specific signaling, and ERRα as the uveal-melanoma-specific NR. In the second *Original research article*, Ma and colleagues analyzed microarray data of differentially expressed genes after the knockdown of the cellular senescence-inhibited gene (CSIG). CSIG, originally identified by this research group, is critical in regulating cell senescence, cell cycle progression, stress response, and tumor metastasis. In this article, they discovered novel CSIG targets that correlate with senescence. Interestingly, they inferred that CSIG regulates the stability of certain target-gene transcripts.

Nath and colleagues presented an interesting *Review* on mitotic proteins in cancer development (8). As the authors pointed out, deregulated transcriptional regulation of mitotic genes are common in cancers, yet mutations are rarely observed for mitotic genes. Cell cycle-related transcription program is interesting and warrants further investigation.

One *Review* and one *Opinion article* appraised the possible use of exosome in early cancer diagnosis (9, 10). In the *Review* article, Qin and colleagues summarized the recent progress on the identification of miRNAs from tumor samples. They grouped different miRNAs based on their location as the cytosolic, body-fluid or exosomal miRNAs. The authors proposed exosomal miRNAs as diagnostic biomarkers for lung tumors. In addition, they described the current methods for the isolation and detection of these RNAs in the tumor samples. Moreover, the authors hypothesized how miRNAs have been transported/released into body fluids from the tumor cells. In accompanying *Opinion article*, Oltra supported cancer-associated exosome in cancer diagnosis and prognosis.

In the end of this Research Topic, Davis and colleagues presented a very interesting *Review* on Nanotetrac in treating cancers (11, 12). In many cancers, Thyroid hormones, T_3_ and T_4_ have pro-angiogenic effects, which might be mediated by α_V_β_3_ integrin. Nanotetrac is a nanoparticulate preparation of a T_4_ substitute, tetrac. Nanotetrac blocks T_4_-triggered α_V_β_3_-mediated transcriptional regulation. Therefore, Nanotetrac might be useful in treating cancers.

By compiling all these excellent manuscripts into one Research Topic, we hope that our readers will find this Research Topic enlightening. We owe our thanks to the staff of Frontiers Endocrinology Office, for their work in the completion of this Research Topic. We are particularly thankful to Davor Vidic, Shaun Evans, Caroline Drage and Byron Bitanihirwe for help in communicating with authors during initiation and completion of the Research Topic. All authors in this Research Topic have provided their broad perspectives on transcriptional regulation, whose insightful thoughts will both benefit the field and be appreciated by fellow researchers. We are indebted to our reviewers/review editors for their contribution to this work.

## Conflict of Interest Statement

The author declare that the research was conducted in the absent of any commercial or financial relationships that could be construed as a potential conflict of interest.
